# Glymphatic dysfunction and cognitive impairment in tuberculous meningitis: insights from diffusion tensor imaging along the perivascular space (DTI-ALPS)

**DOI:** 10.3389/fnins.2026.1764800

**Published:** 2026-03-06

**Authors:** Yilin Wang, Yichuan Wang, Jiaping Liu, Chunsheng Zhao, Shuangshuang Peng, Dailun Hou

**Affiliations:** 1 Department of Radiology, Beijing Tuberculosis and Thoracic Tumor Research Institute, Beijing, China; 2 Department of Radiology, Beijing Chest Hospital, Capital Medical University, Beijing, China

**Keywords:** diffusion tensor imaging, DTI-ALPS index, glymphatic system, mild cognitive impairment, tuberculous meningitis

## Abstract

**Background:**

Tuberculous meningitis (TBM), a severe central nervous system infection, carries significant mortality and long-term neurological morbidity. While cognitive impairment is a common consequences of TBM, the contribution of glymphatic system dysfunction to this process remains poorly characterized.

**Objective:**

To investigate glymphatic function in TBM patients using the diffusion tensor imaging (DTI)-derived along the perivascular space (ALPS) index and evaluate its utility in detecting and predicting mild cognitive impairment (MCI).

**Methods:**

This cross-sectional study enrolled 62 TBM patients and 61 matched healthy controls (HCs). ALPS indices (left/right/whole-brain) were computed from DTI data. Cognitive function was assessed using MMSE, MoCA, TMT-A/B, CDT, VFT, DST, and SDMT. Group comparisons, partial correlation analyses, and ROC curve assessments were performed to examine relationships between glymphatic function and cognitive performance.

**Results:**

TBM patients demonstrated significantly reduced ALPS indices (all *p* < 0.05) and elevated diffusivity in projection (Dyproj) and association (Dzassoc) fiber regions compared to HCs. Within the TBM-MCI subgroup, left Dyproj/Dzassoc correlated negatively with CDT scores (*p* < 0.05), while ALPS indices showed: (1) negative correlations with TMT-A/B (left/whole-brain), (2) positive correlations with SDMT (right/whole-brain). MCI patients exhibited significantly lower right/whole-brain ALPS indices than non-MCI counterparts (*p* < 0.05), with ROC analysis demonstrating moderate predictive value (AUC = 0.70).

**Conclusion:**

The DTI-ALPS index effectively captures glymphatic dysfunction in TBM and correlates with domain-specific cognitive deficits. As a non-invasive biomarker, it shows promise for early identification of TBM patients at risk for MCI, potentially facilitating timely intervention to mitigate cognitive decline.

## 1 Introduction

Tuberculous meningitis (TBM) represents the most severe form of central nervous system tuberculosis, characterised not only by high mortality but also by persistent cognitive impairments such as memory loss, executive dysfunction, and even dementia (Thwaites et al., 2013). Studies have shown that 30%–50% of TBM survivors experience varying degrees of cognitive impairment, significantly affecting their daily functioning and quality of life (Davis et al., 2023; Nimkar et al., 2021). Despite advances in anti-tuberculosis therapy, the mechanisms underlying TBM-related brain injury and cognitive dysfunction remain poorly understood. Notably, mild cognitive impairment (MCI) may be reversible during its early stages (Chang Wong and Chang Chui, 2022). Early identification and timely interventions—such as combining anti-tuberculosis treatment with neuroprotective strategies—may help improve cognitive outcomes in some patients (Da Mesquita et al., 2021; Prasad et al., 2016). Recent research suggests that lymphatic dysfunction, particularly impairment of the glymphatic system and meningeal lymphatic vessels, may play a critical role in the neuropathology of TBM by disrupting cerebrospinal fluid drainage, immune cell trafficking, and the clearance of neurotoxic metabolites (Pinho-Correia et al., 2025).

The glymphatic system, a recently discovered waste clearance pathway in the central nervous system (CNS), is composed of the cerebrospinal fluid (CSF)–interstitial fluid circulation network, aquaporin-4 (AQP4) water channels located on astrocytic endfeet, and meningeal lymphatic vessels. Its primary function is the removal of neurotoxic metabolites, such as β-amyloid (Aβ) and tau proteins, particularly during sleep or under anaesthesia (Iliff et al., 2012; Plog and Nedergaard, 2018). Growing evidence suggests that glymphatic dysfunction is closely associated with the pathogenesis of various neurological disorders, including Aβ accumulation in Alzheimer’s disease (AD), *α*-synuclein aggregation in Parkinson’s disease (PD) (Pang et al., 2024; Iliff et al., 2014), and neuroinflammatory responses following traumatic brain injury (Jessen et al., 2015; Da Mesquita et al., 2021). Furthermore, in infectious meningitis—such as bacterial or viral meningitis—meningeal inflammation-induced fibrosis and impaired CSF circulation may exacerbate glymphatic dysfunction (Louveau et al., 2015; Li et al., 2022). However, this mechanism remains largely unexplored in the context of TBM.

In recent years, the diffusion tensor imaging along the perivascular space (DTI-ALPS) index, derived from diffusion tensor imaging (DTI), has emerged as a novel non-invasive method for evaluating glymphatic function (Liang et al., 2023). This technique quantifies the anisotropic diffusion of water molecules along perivascular spaces, thereby indirectly reflecting the efficiency of the glymphatic system. A higher DTI-ALPS index signifies increased diffusivity of perivascular water, indicating enhanced glymphatic function (Klostranec et al., 2021).

The DTI-ALPS method has demonstrated associations between glymphatic dysfunction and cognitive impairment in various neurological disorders, including PD (Zhao et al., 2025; Zhou et al., 2024), multiple sclerosis (MS) (Bayoumi et al., 2024), acute cerebral hemorrhage (Cai et al., 2025), and AD (Hsu et al., 2023). However, to the best of our knowledge, no studies have yet applied DTI-ALPS to assess glymphatic function in patients with TBM, nor have they examined its relationship with cognitive deficits in this population (Rohlwink et al., 2017; Zhang et al., 2025). Therefore, the present study aims to investigate alterations in glymphatic function in TBM patients using the DTI-ALPS technique and to elucidate the association between glymphatic impairment and the severity of cognitive dysfunction. These findings may provide a theoretical basis for developing treatment strategies to improve cognitive prognosis in TBM.

## 2 Method

### 2.1 Participants

An observational, cross-sectional study was conducted involving 102 participants, including 52 patients with intracranial tuberculosis and 50 healthy controls (HCs). Patients were recruited from the inpatient department of Beijing Chest Hospital, Capital Medical University, while age-, sex-, and education-matched HCs were recruited from the local community. The inclusion criteria for both patients with TBM and HCs were as follows: (1) age 18–60 years; (2) education at primary school level or above; (3) right-handedness; (4) absence of MRI contraindications; and (5) no history of psychiatric or neurological disorders. According to established diagnostic criteria (Jiang et al., 2023; Johansen-Berg, 2007), TBM was diagnosed in patients who met any of the following conditions: with a clinical diagnosis of TBM, i.e., at least 5 days of meningitis symptoms, and cerebrospinal fluid (CSF) abnormalities; with anti-tuberculosis chemotherapy already started or planned by the treating clinician. All enrolled patients were in the acute phase of TBM, characterized by newly diagnosed, untreated status at presentation. The median time from the onset of meningitis symptoms to study enrollment was 1 week (range 1 days to 2 weeks).

The general exclusion criteria for patients with TBM and HCs were as follows: (1) a history of substance (drug, nicotine, or alcohol) abuse; (2) pregnancy or lactation; (3) concomitant neurological, cardiovascular, cerebrovascular, or endocrine disorders; (4) infection with human immunodeficiency virus; (5) hearing, visual, or physical impairments that precluded completion of cognitive or imaging assessments; and (6) poor MRI data quality (e.g., significant susceptibility artifacts or incomplete raw data). This study was conducted in accordance with the Declaration of Helsinki (2013 revision) and was approved by the Ethics Committee of Beijing Chest Hospital of Capital Medical University (BJXK-2024-KY-16). All participants provided informed consent.

Based on previously established diagnostic criteria (Thwaites et al., 2009; Yadav et al., 2010), patients were diagnosed with intracranial tuberculosis if they met either of the following: (1) a positive result for acid-fast bacilli in CSF, and/or a positive *Mycobacterium tuberculosis* (MTB) culture in CSF, and/or a positive commercial nucleic acid amplification test in CSF; or (2) isolation of MTB from a site outside the central nervous system, along with clinical features indicative of intracranial tuberculosis and typical CSF findings, including pleocytosis (>20 cells/μL), lymphocyte predominance (>60%), protein concentration >1 g/L, a CSF-to-blood glucose ratio <0.6, and negative results on India ink staining and cytological tests for malignant cells. All patients underwent CSF analysis.

The general exclusion criteria for both patients with intracranial tuberculosis and HCs were as follows: (1) a history of drug, nicotine, or alcohol abuse; (2) pregnancy or breastfeeding; (3) comorbid neurological, cardiovascular, cerebrovascular, or endocrine disorders; (4) human immunodeficiency virus (HIV) infection; (5) hearing, visual, or physical impairments that could interfere with test completion; (6) poor-quality MRI data due to significant susceptibility artifacts or incomplete acquisition. This study was conducted by the Declaration of Helsinki (as revised in 2013) and was approved by the Ethics Committee of our hospital (BJXK-2024-KY-16). Informed consent was obtained from all participants.

### 2.2 Cognitive assessments

All participants completed a series of cognitive assessments in a quiet room within the radiology department. These tests were administered by trained researchers to ensure procedural consistency. The Mini-Mental State Examination (MMSE) and the Montreal Cognitive Assessment (MoCA) were used to evaluate overall cognitive function (Nasreddine et al., 2005; Folstein et al., 1975). The Trail Making Test Parts A and B (TMT-A and TMT-B) assessed executive function, attention, and cognitive flexibility (Bowie and Harvey, 2006). Visuospatial abilities were evaluated using the Clock Drawing Test (CDT) (Shulman, 2000). The Verbal Fluency Test (VFT) was administered to examine verbal ability and executive control (Lezak et al., 2004). The Digit Span Test (DST) was used to measure attention and working memory (Lezak et al., 2004). Finally, the Symbol Digit Modalities Test (SDMT) was employed to assess information processing speed (Lezak et al., 2004).

MCI grouping criteria were based on scores from the MMSE and the MoCA, both of which have a maximum score of 30. For participants with fewer than 12 years of education, one point was added to the MoCA score in accordance with standard guidelines. Participants were classified as having MCI if they scored <26 on the MoCA and between 21 and 26 on the MMSE. Those with MMSE scores >27 and MoCA scores ≥26 were considered to have normal cognitive function. In cases where MMSE and MoCA scores were inconsistent, the MMSE score was prioritized, and clinical features of MCI were taken into account. These included:

(1) Reports from family members or other informants indicating memory loss as the primary complaint, with other cognitive functions either intact or only mildly impaired;
(2) Preserved activities of daily living that did not meet the diagnostic criteria for dementia; and
(3) Exclusion of other neurological or systemic diseases that could account for cognitive decline.

Based on the cognitive assessment results, patients with intracranial tuberculosis were categorized into two subgroups: TBM-MCI and TBM-nonMCI.

### 2.3 MRI data acquisition

MRI scanning was performed using a 3T Signa Architect (GE Healthcare) with a 48-channel phased-array head coil after neurocognitive tests. High-resolution 3D-T1 magnetization prepared rapid gradient echo (MPRAGE) images field-of-view (FOV): 240 × 240 mm^2^, voxel size: 0.9 × 0.9 × 0.9 mm^3^, slice thickness = 0.6 mm, number of slices = 484, layer spacing = 0, repetition time (TR): 7.9 ms, flip angle = 8^°^, scanning time = 7 min 18 s, used for anatomical registration, were acquired at the start of each scanning session.

The DTI scanning parameters were same for both scanners as shown below: FOV = 240 × 240 mm^2^, TR = 8,000 ms, echo time = minimum, matrix = 128 × 130, number of diffusion encoding directions = 30, slice thickness = 5 mm, number of slices = 29, layer spacing = 0, and gradient values b = 0 s/mm^2^ and b = 1,000 s/ mm^2^, scanning time = 4 min 16 s. In addition, T2-weighted fluid attenuated inversion recovery (T2-FLAIR) images of all patients, to exclude brain diseases such as stroke or tumors, with the parameters: TR = auto, TE = minimum, FOV = 240 × 240 mm^2^, slice thickness = 5 mm, number of slices = 29, layer spacing = 0, and gadolinium enhanced T1-weighted (Gd-T1) images of 62 patients were collected to better characterize the brain lesions for classifying the types of intracranial tuberculosis and to assess the existence of the ventricle enlargement.

Total intracranial volume was processed from the whole-brain T1-weighted images segmentation using the Computational Anatomy Toolbox 12 (CAT-12), a toolbox of Statistical Parametric Mapping version 12 software package (SPM-12, https://www.fil.ion.ucl.ac.uk/spm/software/spm12/).

### 2.4 Processing steps for DTI-ALPS

**Figure 1:**
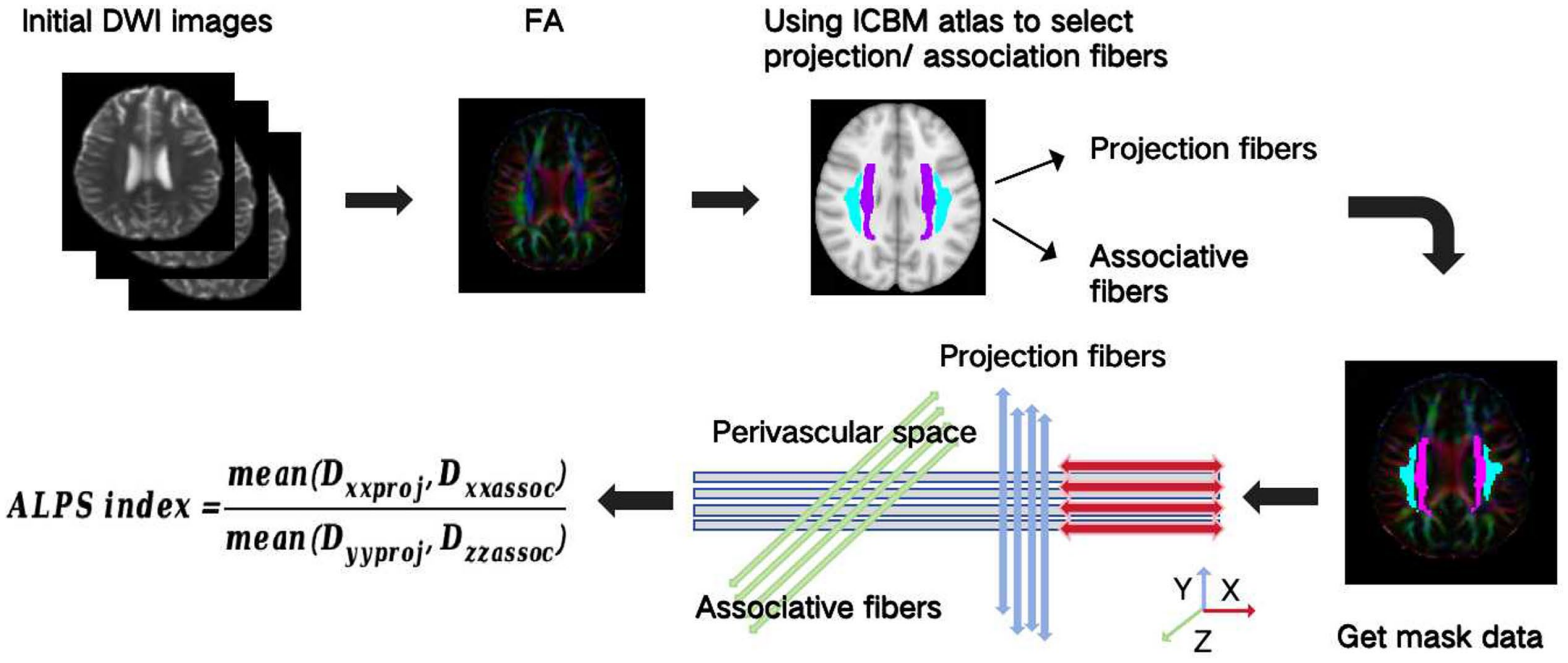
Analysis process from initial diffusion-weighted imaging (DWI) images to calculate analysis along the perivascular space (ALPS) indexes.

The workflow for DTI-ALPS calculation is illustrated in Figure 1. The DTI-ALPS method enables the quantification of glymphatic activity along perivascular spaces by analysing multidirectional diffusivity maps derived from DTI data. The DTI images were processed and modelled using the FMRIB Software Library (FSL, http://www.fmrib.ox.ac.uk/fsl/), following these steps.

#### 2.4.1 Anterior commissure–posterior commissure (AC-PC) alignment and reorientation

To correct for potential head tilt in raw images, the b0 image was rigidly aligned to a standard template using FSL’s FLIRT tool (six degrees of freedom: rotation and translation) (Jenkinson et al., 2012), ensuring AC–PC alignment. The resulting linear deformation field was then applied to adjust the diffusion gradient directions accordingly.

#### 2.4.2 Skull stripping and brain extraction

Non-brain tissues (e.g., scalp, skull) were removed using FSL’s Brain Extraction Tool (BET) (Smith, 2002), generating a brain mask from the b0 image for subsequent processing.

#### 2.4.3 Preprocessing and denoising

Diffusion-weighted images (DWI) were preprocessed using MRtrix3 (Tournier et al., 2019) and FSL. The processing steps included: PCA-based denoising to reduce random noise, Gibbs ringing correction to suppress truncation artifacts; eddy current and motion correction using FSL’s *eddy* tool (Andersson and Sotiropoulos, 2016); and ANTs-based N4 bias field correction (Tustison et al., 2010) to address B₀ inhomogeneity. Following motion correction, head motion was quantified using the mean framewise displacement (FD). To minimize the impact of excessive motion on diffusion metrics, subjects with a mean FD > 0.5 mm were excluded from subsequent analysis, in line with conservative practices in the field. The eddy tool’s quality control reports were also visually examined for all subjects.

#### 2.4.4 Diffusion tensor reconstruction

The diffusion tensor model was fitted using FSL’s dtifit (Smith, 2002), producing maps of fractional anisotropy (FA) and principal directional diffusivities (Dxx, Dyy, Dzz).

#### 2.4.5 ALPS calculation

FA maps were registered to the ICBM DTI-81 standard space atlas via T1WI using FSL, with registration accuracy verified by visual inspection. The ICBM DTI-81 atlas contains labels for projection fibers (e.g., superior and posterior corona radiata) and association fibers (e.g., superior longitudinal fasciculus) in the periventricular region. Projection and association fibers within 25–33 mm above the AC–PC line in MNI space were extracted, as this region corresponds to the *x*-axis direction of penetrating vessels in the deep white matter. The ALPS index and diffusivity values for projection and association fibers were computed based on the method described by Taoka et al. (2017).

### 2.5 Procedure for ROI placement

The ROIs for calculating the ALPS index were placed using a semi-automated, atlas-guided method to ensure consistency. The specific procedure was as follows:

Anatomical level and slice selection: All analyses were performed on axial slices at the level of the body of the lateral ventricle in MNI space. This level was chosen as it reliably contains the periventricular projection and association fibers relevant to the ALPS index calculation.

Initial ROI generation: Based on the registered ICBM DTI-81 atlas, initial ROIs for the projection fibers (primarily encompassing the superior and posterior corona radiata) and association fibers (primarily encompassing the superior longitudinal fasciculus) were automatically extracted within the pre-defined zone of 25–33 mm above the AC–PC line.

Manual verification and refinement: These atlas-derived ROIs were then visually inspected and manually refined on each individual’s FA map by a trained researcher (with 10 years of experience). Refinement ensured that the ROIs accurately outlined the target white matter tracts while excluding cerebrospinal fluid in the lateral ventricles and adjacent grey matter. This step was guided by the clear anatomical landmarks of the lateral ventricles and the directional orientation of the fiber tracts themselves.

Calculation: Finally, the mean diffusivity along the x-axis (Dxx) within the refined projection fiber ROI and along the y-axis (Dyy) within the refined association fiber ROI were computed: The ALPS index was calculated as: ALPS index = mean (Dxxproj, Dxxassoc) / mean (Dyyproj, Dzzassoc).

### 2.6 Statistical analyses

In SPSS 30.0 (IBM Corp., Armonk, NY, USA), with a significance at *p* < 0.05, the clinical and cognitive function, and diffusivities, were compared using a t-test for continuous variables, Mann–Whitney U-test for ordinal variables, and chi-square test for categorical variables. Normally distributed continuous variables are presented as mean (standard deviation); non-normally distributed variables are presented as median (first quartile, third quartile).

The group correlation with ALPS index and cognitive function in patients with TBM was estimated using partial correlation analyses, controlling for age, sex and education, while differences between TBM-MCI and TBM-nonMCI groups were estimated using analysis of covariance (ANCOVA). Differences in means from the independent samples *t*-test were performed to determine the mean differences, effect sizes as quantified by Cohen’s *d* (d), and 95% confidence intervals (CIs) calculated from 5,000 bootstraps. Diagnostic accuracy was determined through receiver operating characteristic (ROC) curve analysis, with performance quantified by the area under the curve (AUC).

## 3 Results

### 3.1 Demographics and cognitive functions

**Figure 2:**
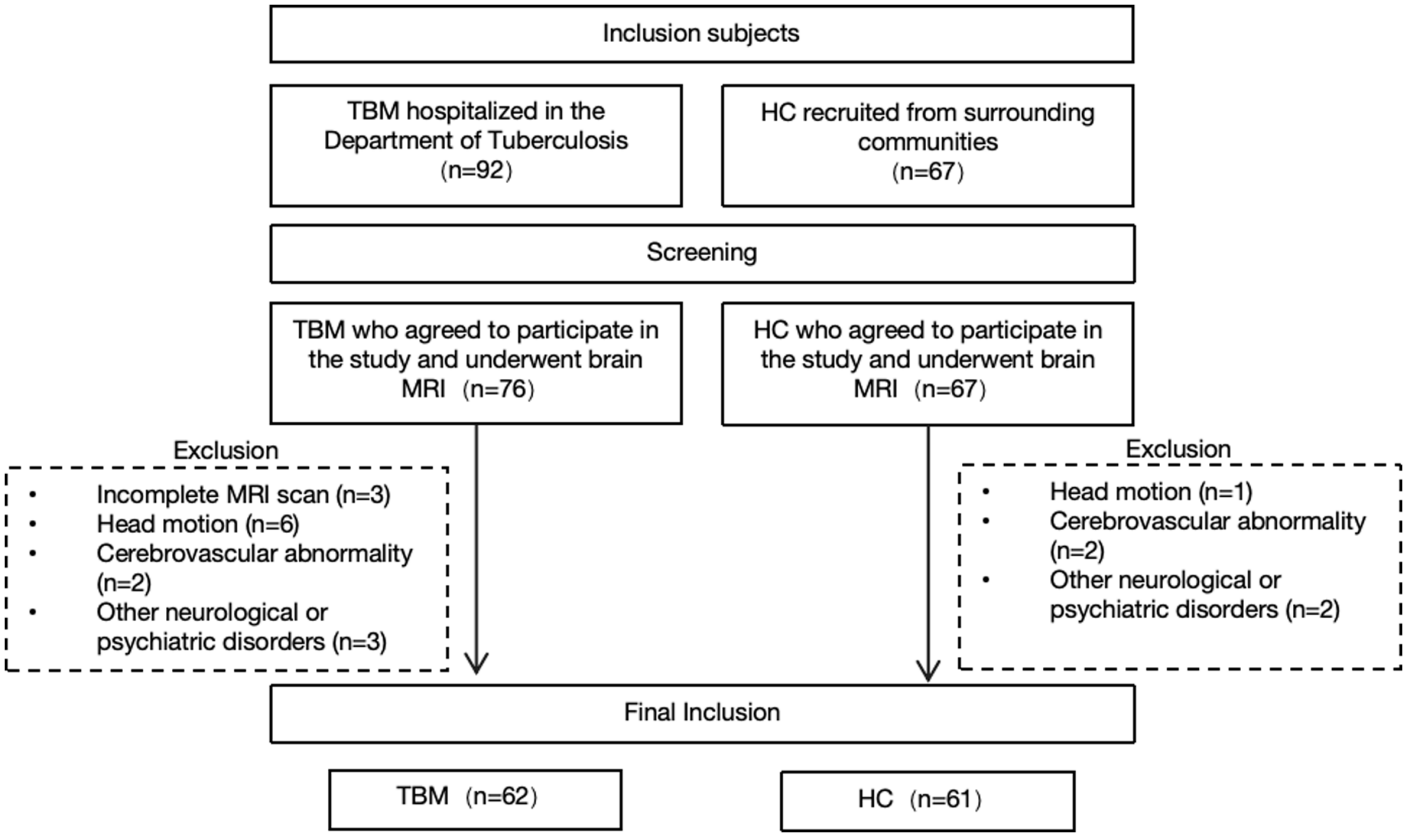
Flowchart of the participant inclusion process.

The study finally included 62 patients with intracranial TB and 61 HCs (Figure 2). As shown in Table 1, there were no significant differences between the TBM and HCs groups in terms of age, gender, years of education, DST, or TIV. However, cognitive performance was significantly lower in the TBM group. Specifically, general cognitive function (MMSE, MoCA), executive function, attention, and cognitive flexibility (TMT; *p* < 0.05), visuospatial abilities (CDT), verbal fluency, memory, and executive control (VFT), as well as attention, tracking ability, and information processing speed (SDMT), were all significantly reduced compared to the HC group (*p* < 0.001).

**Table 1:** Demographics and clinical characteristics of patients with TBM and HCs.

Demographics	TBM (*n* = 62)	HCs (*n* = 61)	*p* value
Age (year)	42.34 (14.70)	43.11 (14.82)	0.771
Gender (male/female)	38/24	32/29	0.323
Education (year)	12.34 (3.03)	13.13 (2.99)	0.147
MMSE	26 (25, 28.25)	30 (29, 30)	<0.001^*^
MoCA	23.61 (4.08)	26.92 (2.78)	<0.001^*^
TMT-A (s)	48.50 (34, 63)	33 (25, 44)	<0.001^*^
TMT-B (s)	103.50 (75, 161.50)	81 (64, 115)	0.020^*^
CDT	5 (4, 5)	5 (5, 5)	0.001^*^
VFT	35.19 (8.72)	44.80 (11.63)	<0.001^*^
DST forward	61 (9, 12)	10.89 (2.56)	0.597
DST backward	61 (6, 9.25)	8 (5.50, 10)	0.189
SDMT	39.37 (10.71)	50.44 (13.29)	<0.001^*^
TIV (cm^3^)	1447.91 (130.52)	1438.20 (130.98)	0.434

Normal distribution data are presented as mean (SD); non-normal distribution data are presented as median (first quartile, third quartile); HCs, healthy controls; MCI, mild cognitive impairment; MMSE, mini-mental state examination; MoCA, montreal cognitive assessment; TMT, trail making test; CDT, clock drawing test; VFT, verbal fluency test; DST, digital span test; SDMT, symbol-digit modalities test; TIV, total intracranial volume; *p < 0.05.

### 3.2 Comparison of diffusion coefficients between the two groups

**Figure 3:**
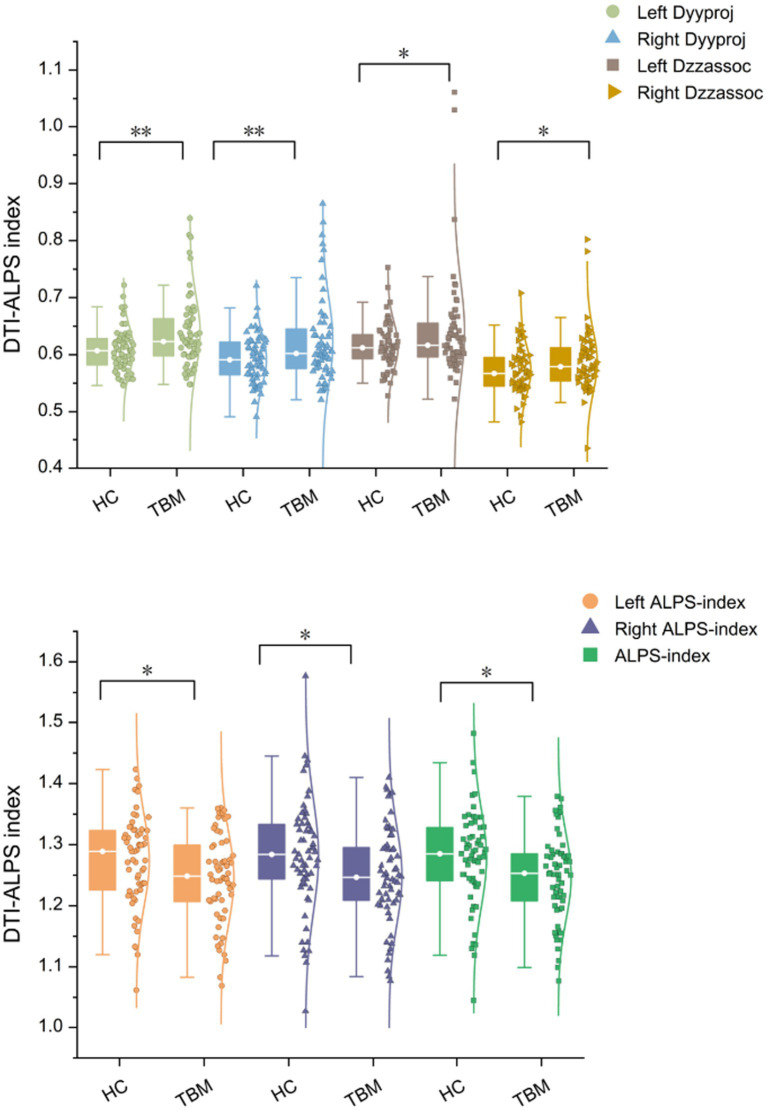
Distribution of the ALPS-index in TBM and HCs group. ALPS, Diffusion tensor imaging along the perivascular space; Dyyproj, diffusivity along the *y*-axis in projection fiber area; Dzzassoc, diffusivity along the *z*-axis in association fiber area; diffusivity was presented as apparent diffusion coefficient values (× 10^−3^ mm^2^/s). *p < 0.05, **p < 0.01.

As shown in Table 2, after adjusting for age, sex, and years of education, the left and right ALPS indices, as well as the whole-brain ALPS index, were significantly lower in the TBM group compared to the NC group. In contrast, the diffusivity in the bilateral projection fiber areas along the y-axis (Dyyproj) and in the bilateral association fiber areas along the z-axis (Dzzassoc) was significantly higher in the TBM group (Figure 3). No significant differences were observed in Dxxproj and Dxxassoc between the two groups.

**Table 2:** Comparison of the diffusivities between TBM patients and HCs.

Diffusivity	TBM (*n* = 62)	HCs (*n* = 61)	*p* value
Left Dxxproj	0.79 (0.76, 0.82)	0.79 (0.04)	0.359
Left Dyyproj	0.62 (0.60, 0.66)	0.61 (0.58, 0.63)	0.003*
Left Dxxassoc	0.78 (0.75, 0.81)	0.77 (0.05)	0.077
Left Dzzassoc	0.62 (0.60, 0.66)	0.61 (0.04)	0.049*
Right Dyyproj	0.60 (0.58, 0.65)	0.59 (0.04)	0.006*
Right Dxxproj	0.76 (0.73, 0.80)	0.76 (0.04)	0.130
Right Dxxassoc	0.73 (0.70, 0.76)	0.73 (0.05)	0.677
Right Dzzassoc	0.58 (0.55, 0.61)	0.57 (0.04)	0.049*
Left ALPS-index	1.25 (0.07)	1.27 (0.07)	0.035*
Right ALPS-index	1.25 (0.08)	1.28 (0.09)	0.036*
ALPS-index	1.25 (0.07)	1.28 (0.08)	0.023*

Normal distribution data are presented as mean (SD); non-normal distribution data are presented as median (first quartile, third quartile); HCs, healthy controls; MCI, mild cognitive impairment; Dxxassoc/Dzzassoc, diffusivity along the *x*-axis/*z*-axis in association fiber area; Dyyproj/Dxxproj, diffusivity along the *y*-axis/*x*-axis in projection fiber area; diffusivity was presented as apparent diffusion coefficient values (× 10^−3^ mm^2^/s); *p < 0.05.

### 3.3 Correlations between DTI-ALPS and cognitive function

**Figure 4:**
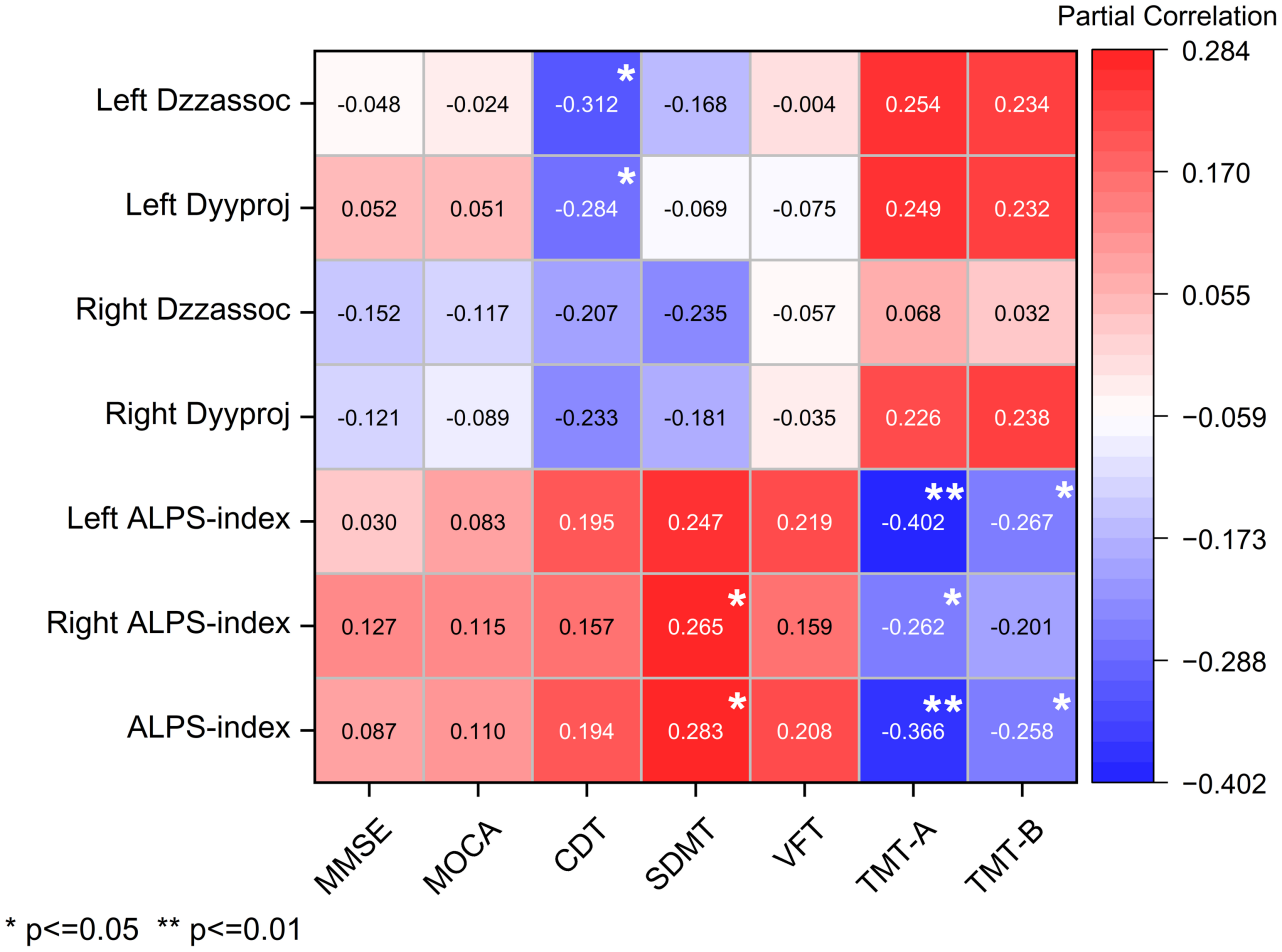
Correlation between ALPS index and cognitive performance in TBM and HCs groups.

As shown in Figure 4, correlations were observed between DTI-ALPS indices and cognitive performance. After adjusting for age, gender, and years of education, the following significant relationships were identified: The left Dzzassoc index was negatively correlated with visuospatial function as measured by the CDT (*r* = −0.312, *p* = 0.016). The left Dxxproj index was also negatively correlated with CDT scores (*r* = −0.270, *p* = 0.038). The left Dyyproj index showed a negative correlation with CDT scores (*r* = −0.284, *p* = 0.029). The left ALPS index was negatively correlated with executive function, attention, and cognitive flexibility as assessed by TMT-A and TMT-B (*r* = −0.402, *p* = 0.002; *r* = −0.267, *p* = 0.041, respectively). The right ALPS index was positively correlated with attention, tracking ability, and information processing speed (SDMT: *r* = 0.265, *p* = 0.043) and negatively correlated with TMT-A (*r* = −0.262, *p* = 0.045). The whole-brain ALPS index was positively correlated with SDMT (*r* = 0.283, *p* = 0.030) and negatively correlated with TMT-A and TMT-B (*r* = −0.366, *p* = 0.004; *r* = −0.258, *p* = 0.048, respectively). These findings suggest that higher ALPS indices are associated with better cognitive performance in patients with MCI. In contrast, increased diffusivity in bilateral projection (Dyyproj) and association (Dzzassoc) fiber regions was associated with poorer cognitive outcomes, implying that preserved glymphatic function may play a protective role in cognition.

### 3.4 Comparison between TBM patients with and without MCI

#### 3.4.1 Comparison of demographics and cognitive function

Table 3 summarizes the demographic and cognitive characteristics of TBM-MCI (defined as MoCA < 26 and MMSE 21–26) and TBM-nonMCI. There were no statistically significant differences between the MCI and non-MCI groups in terms of age, gender, or TIV. However, TBM-MCI showed significantly lower performance on general cognitive assessments (MMSE, MoCA), executive function and attention (TMT), visuospatial ability (CDT), attention and working memory (DST), and information processing speed (SDMT), compared to TBM-nonMCI. No significant differences were found in VFT scores between the two groups.

**Figure 5:**
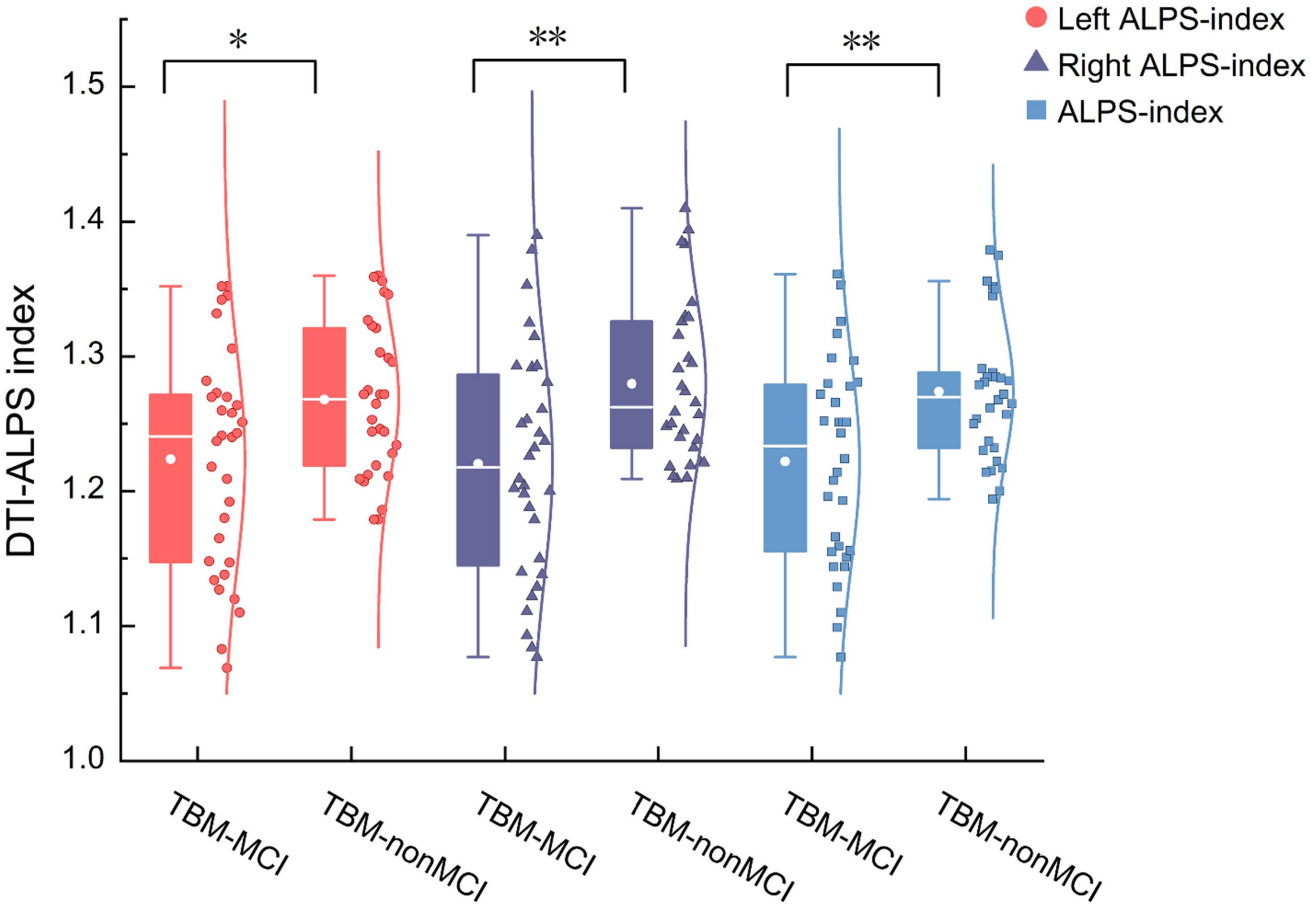
Distribution of the ALPS-index in TBM-MCI and TBM-nonMCI group. ALPS, diffusion tensor imaging along the perivascular space; diffusivity was presented as apparent diffusion coefficient values (× 10^−3^ mm^2^/s). *p < 0.05, **p < 0.01.

Table 4 summarizes the diffusivities of TBM-MCI and TBM-nonMCI. The left, right, and whole-brain ALPS indices were significantly lower in TBM-MCI compared to TBM-nonMCI (1.22 vs.1.27, *p* = 0.015, 1.22 vs.1.26, *p* = 0.002, and 1.22 vs.1.27, *p* = 0.002, respectively) (Figure 5, see cloud and rain charts within the group).

**Table 3:** Demographics and clinical characteristics of TBM-MCI and TBM-nonMCI.

Demographics	TBM (*n* = 62)	TBM-MCI (*n* = 32)	TBM-nonMCI (*n* = 30)	*p* value
Age (year)	42.34 (14.70)	44.97 (15.16)	39.53 (13.89)	0.147
Gender (male/female)	38/24	20/12	18/12	0.840
Education (year)	12.34 (3.03)	10 (10, 13)	13.00 (10, 17)	<0.001*
MMSE	26 (25, 28.25)	25 (23, 26)	29 (28, 29)	<0.001*
MoCA	23.61 (4.08)	23 (21, 24)	26 (25, 27)	<0.001*
TMT-A (s)	48.50 (34, 63)	63.69 (21.26)	38.00 (28.75, 44.50)	<0.001*
TMT-B (s)	103.50 (75, 161.50)	137 (99.75, 217.50)	79.00 (66, 108.25)	<0.001*
CDT	5 (4, 5)	4 (4, 5)	5 (5, 5)	0.006*
VFT	35.19 (8.72)	33.09 (6.53)	37.43 (10.22)	0.054
DST forward	61 (9, 12)	9.59 (1.97)	12.00 (11.00, 13.00)	<0.001*
DST backward	61 (6, 9.25)	6 (4.25, 7.00)	9.50 (7.00, 11.00)	<0.001*
SDMT	39.37 (10.71)	32.75 (8.18)	46.43 (8.34)	<0.001*
TIV (cm^3^)	1447.91 (130.52)	1428.75 (114.41)	1468.35 (144.93)	0.236

Normal distribution data are presented as mean (SD); non-normal distribution data are presented as median (first quartile, third quartile); HCs, healthy controls; MCI, mild cognitive impairment; MMSE, mini-mental state examination; MoCA, montreal cognitive assessment; TMT, trail making test; CDT, clock drawing test; VFT, verbal fluency test; DST, digital span test; SDMT, symbol-digit modalities test; TIV, total intracranial volume; *p < 0.05.

**Table 4:** Comparison of the diffusivities between TBM-MCI and TBM-nonMCI.

Diffusivity	TBM (*n* = 62)	TBM-MCI (*n* = 32)	TBM-nonMCI (*n* = 30)	*p* value
Left Dxxproj	0.79 (0.76, 0.82)	0.79 (0.75, 0.83)	0.80 (0.06)	0.961
Left Dyyproj	0.62 (0.60, 0.66)	0.63 (0.60, 0.68)	0.62 (0.60, 0.66)	0.592
Left Dxxassoc	0.78 (0.75, 0.81)	0.78 (0.75, 0.82)	0.78 (0.04)	0.978
Left Dzzassoc	0.62 (0.60, 0.66)	0.63 (0.60, 0.69)	0.62 (0.04)	0.066
Right Dyyproj	0.60 (0.57, 0.65)	0.61 (0.57, 0.67)	0.60 (0.58, 0.62)	0.394
Right Dxxproj	0.76 (0.73, 0.80)	0.76 (0.74, 0.80)	0.78 (0.06)	0.693
Right Dxxassoc	0.73 (0.70, 0.76)	0.71 (0.69, 0.76)	0.73 (0.04)	0.147
Right Dzzassoc	0.58 (0.55, 0.61)	0.58 (0.56, 0.63)	0.58 (0.55, 0.60)	0.098
Left ALPS-index	1.25 (0.07)	1.22 (0.08)	1.27 (0.06)	0.015*
Right ALPS-index	1.25 (0.08)	1.22 (0.09)	1.26 (1.23, 1.33)	0.002*
ALPS-index	1.25 (0.07)	1.22 (0.08)	1.27 (0.05)	0.002*

Normal distribution data are presented as mean (SD); non-normal distribution data are presented as median (first quartile, third quartile); MCI, mild cognitive impairment; Dxxassoc/Dzzassoc, diffusivity along the *x*-axis/*z*-axis in association fiber area; Dyyproj/Dxxproj, diffusivity along the *y*-axis/*x*-axis in projection fiber area; diffusivity was presented as apparent diffusion coefficient values (× 10^−3^ mm^2^/s); *p < 0.05.

#### 3.4.2 Differences in DTI-ALPS index and cognitive function between MCI and non-MCI groups

As shown in Table 5, ANCOVA adjusting for age, gender, and years of education revealed significant differences in ALPS index and cognitive function (MMSE, MoCA, TMT, DST, SDMT) between the MCI and non-MCI groups. Specifically, the right ALPS index was significantly lower in the MCI group compared to the non-MCI group (mean difference = −0.06; 95% CI [−0.10, −0.02]; *p* = 0.022; Cohen’s d = −0.81; 95% CI [−1.32, −0.29]). Similarly, the whole-brain ALPS index was also significantly reduced in the MCI group (mean difference = −0.05; 95% CI [−0.09, −0.02]; *p* = 0.019; Cohen’s d = −0.81; 95% CI [−1.32, −0.29]). The mean differences between groups are presented in Table 6.

**Table 5:** ANCOVA between the diffusivities and cognitive function for TBM-MCI and TBM-nonMCI.

Dependent variable	F	*p* value	η^2^p effect size
Left ALPS-index	3.05	0.086	0.05
Right ALPS-index	6.41	0.014*	0.10
ALPS-index	5.69	0.020*	0.09
MMSE	30.29	0.000*	0.35
MOCA	13.35	0.001*	0.19
TMT-A	14.55	0.000*	0.20
TMT-B	16.48	0.000*	0.22
CDT	2.83	0.098	0.05
DST-F	18.23	0.000*	0.24
DST-B	39.27	0.000*	0.41
SDMT	38.57	0.000*	0.40

*p < 0.05.

**Table 6:** Adjusted group differences in diffusivities and cognitive function between TBM-MCI and TBM-nonMCI.

Diffusivity	*t*	MD (95% CI)	*p* value	Cohen’s *d* (95% CI)
Right ALPS-index	−3.18	−0.06 (−0.10, −0.02)	0.002	−0.81 (−1.32, −0.29)
ALPS-index	−3.18	−0.05 (−0.09, −0.02)	0.002	−0.80 (−1.31, −0.28)
MMSE	−7.19	−4.93 (−6.32, −3.54)	0.000	−1.78 (−2.36, −1.18)
MOCA	−5.19	−4.88 (−6.76, −3.00)	0.000	−1.32 (−1.87, −0.76)
TMT-A	4.42	23.72 (12.99, 34.45)	0.000	1.12 (0.58, 1.66)
TMT-B	4.28	66.82 (35.54, 98.10)	0.000	1.08 (0.54, 1.61)
DST-F	−5.43	−2.27 (−3.11, −1.43)	0.000	−1.36 (−1.91, −0.80)
DST-B	−7.31	−3.26 (−4.15, −2.36)	0.000	−1.88 (−2.47, −1.27)
SDMT	−6.52	−13.68 (−17.88, −9.49)	0.000	−1.66 (−2.23, −1.07)

MD, mean difference, Covariates: age, sex, and education years.

#### 3.4.3 Prediction of cognitive dysfunction using ALPS index

**Figure 6:**
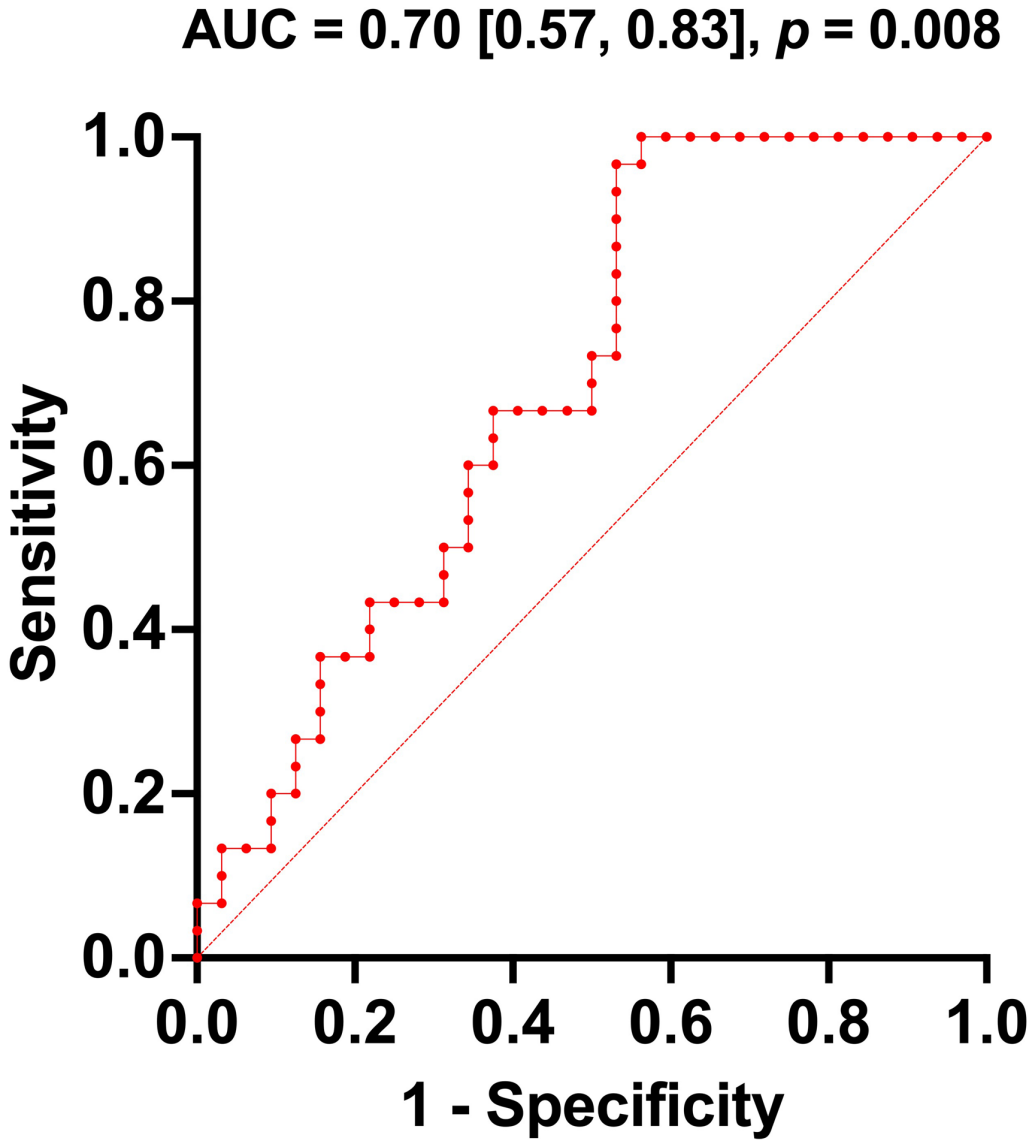
Multivariable logistic regression receiver operating curve. ROC curve assessing the combined performance of the right DTI-ALPS index and mean DTI-ALPS index in predicting MCI level.

A multivariate logistic regression analysis was conducted to assess the predictive value of the right ALPS index and the whole-brain ALPS index for identifying comorbid MCI in TBM patients. The model yielded an AUC of 0.70 (95% CI: 0.57–0.83; *p* = 0.008), with a Youden’s index of 0.44, indicating moderate predictive accuracy. The ROC curve is presented in Figure 6.

## 4 Discussion

In this study, we employed the DTI-ALPS index as a surrogate marker of glymphatic clearance function to investigate the relationship between glymphatic dysfunction and cognitive performance in patients with TBM. Furthermore, we constructed a predictive model using DTI-ALPS to serve as a potential biomarker for identifying and predicting cognitive impairment in TBM patients. Our findings demonstrated that the DTI-ALPS index was significantly lower in the TBM group compared to healthy controls, indicating impaired glymphatic function in TBM. Notably, this overall reduction in glymphatic activity was associated with cognitive deficits. Meanwhile, diffusivity in the projection and association fibers was increased in the TBM group compared to controls. Moreover, the DTI-ALPS index effectively distinguished TBM patients with MCI from those with normal cognition. To the best of our knowledge, this is the first study to demonstrate an association between glymphatic dysfunction and cognitive impairment in TBM. Unlike previous research, which lacked a mechanistic explanation, our findings provide novel insight into the pathophysiological processes underlying TBM-related cognitive decline. These results suggest that glymphatic impairment may contribute to the development of cognitive dysfunction in TBM and highlight its potential as a predictive biomarker and therapeutic target for neuroprotection in this population.

Traditional assessments of lymphatic function rely on invasive contrast agents, which limit their clinical applicability. In recent years, the introduction of the DTI-ALPS index has addressed this limitation. This noninvasive technique enables the quantitative evaluation of glymphatic system function by measuring water diffusivity along the perivascular space (Naganawa and Taoka, 2022). Previous studies have identified a relationship between cognitive impairment and glymphatic dysfunction in MS (Bayoumi et al., 2024), AD (Huang S. Y. et al., 2024), frontotemporal dementia (FTD) (Jiang et al., 2023), and PD (Pang et al., 2024). These conditions are commonly associated with impaired clearance of neurotoxic substances—such as Aβ, tau, or inflammatory mediators—and dysfunction of aquaporin-4 (AQP4) (Iliff et al., 2014; Kamagata et al., 2022; Tang et al., 2022; McMurray et al., 2016; Rehman and Akash, 2017).

Therefore, consistent with previous studies, our findings also indicate that glymphatic function is reduced in TBM patients and decreases further in those with cognitive impairment. Given the high mortality of TBM and the increasing prevalence of cognitive dysfunction among survivors, considerable research has focused on identifying MRI-based biomarkers for early detection and prevention of TBM-related cognitive decline. For example, cortical thickness on T1WI (Shin et al., 2021) and white matter structural connectivity assessed by DTI (Huang X. et al., 2024) have been proposed as predictors of cognitive progression in PD. Similarly, glymphatic dysfunction evaluated via the DTI-ALPS index may serve as an additional MRI biomarker for predicting cognitive impairment in TBM patients and as a potential target for therapeutic intervention.

In our study, the left ALPS index, right ALPS index, and overall ALPS index were significantly reduced in patients with tuberculous meningitis (TBM), and these reductions were positively correlated with attention, tracking ability, information processing speed, executive function, and cognitive flexibility. Additionally, the left Dzz (association fiber) and left Dyy (projection fiber) indices were positively correlated with visuospatial function. The significant decrease in the DTI-ALPS index in TBM suggests impaired glymphatic function. This dysfunction may result from multiple pathological mechanisms, primarily including inflammation-induced disruption of AQP4 polarity and meningeal lymphatic fibrosis. The chronic inflammatory environment in TBM—characterized by elevated levels of TNF-α and IL-1β—can interfere with the polarized localization of AQP4 channels on astrocytic endfeet, thereby impairing glymphatic clearance function (Iliff et al., 2012; Mestre et al., 2018). Inflammation can also lead to increased blood–brain barrier (BBB) permeability, enabling plasma proteins such as fibrinogen to bind to integrin receptors (αVβ3) on astrocytes, initiating neurotoxic cascades that further inhibit AQP4 function (Mestre et al., 2018; Petersen et al., 2017; Merlini et al., 2019; Mi et al., 2025). Notably, mycobacterial components such as lipoarabinomannan (LAM) may activate microglia through Toll-like receptor (TLR) signaling pathways, leading to the release of reactive oxygen species (ROS) and subsequent downregulation of AQP4 expression (Kress et al., 2014).

On the other hand, characteristic granuloma formation and fibrotic thickening of the meninges in TBM may directly compress meningeal lymphatic vessels, obstructing CSF drainage (Louveau et al., 2015; Manyelo et al., 2021; Weller et al., 2018). Upregulation of the profibrotic TGF-β signaling pathway promotes abnormal perivascular deposition of type I/III collagen, reducing vessel wall compliance (Da Mesquita et al., 2021; Hitpass Romero et al., 2025; Moinuddin and Tada, 2000). Lymphatic dysfunction further exacerbates neuroinflammation and the accumulation of pathological proteins. Infiltrating CD4^+^ T cells and macrophages can secrete proteolytic enzymes such as MMP-9 (Si et al., 2024), which degrade endothelial junction proteins and disrupt the structural integrity of lymphatic vessels (Louveau et al., 2018). These changes collectively impair the clearance of neurotoxic substances such as Aβ and tau, thereby contributing to neuronal damage and cognitive decline. Furthermore, a postmortem study of pneumococcal meningitis revealed that the degree of meningeal fibrosis was associated with lower cognitive performance before death, as assessed by tests such as the MMSE (van de Beek et al., 2016).

In this study, we used DTI-ALPS to assess glymphatic function in TBM patients for the first time and found a significant association with cognitive impairment (left ALPS index,1.25 vs.1.27, *p* = 0.035, right ALPS index,1.25 vs.1.28, *p* = 0.036, ALPS index, 1.25 vs.1.28, *p* = 0.023). We also observed increased diffusivity in TBM patients compared to HCs in both projection fibres (Dyy), with increases of 1.64% on the left and 1.69% on the right (*p* < 0.05), and association fibres (Dxx), with increases of 1.64% on the left and 1.75% on the right (*p* < 0.05). These findings provide new insights into the neuropathological mechanisms underlying TBM.

One possible explanation is that these changes reflect a compensatory response of the nervous system to chronic injury. Animal studies have shown that in models of CNS inflammation, long-range fiber tracts such as the corticospinal tract may undergo axonal sprouting and synaptic remodeling, while short-range association fibers are more vulnerable to damage (Werring et al., 2000). Second, meningeal fibrosis—a hallmark of TBM—may alter the microenvironment of water diffusion, leading to artifactually increased diffusivity values. Additionally, chronic inflammation may trigger neuroplastic responses, including reinforcement of specific fiber tracts and relative preservation of others due to selective neuronal loss. These changes may partly compensate for the reduced network efficiency caused by glymphatic dysfunction (Johansen-Berg, 2007).

These findings suggest that TBM treatment should target both glymphatic restoration (e.g., anti-inflammatory therapy) and white matter protection (e.g., neurotrophic support). Notably, similar to findings by Budde et al. (2008) in MS models, DTI can sensitively detect microstructural alterations in white matter. However, elevated FA values in TBM may reflect atypical pathological processes such as fibrosis or selective remodeling, rather than classical axonal damage. This warrants further subgroup analyses in TBM patients.

The observed dissociation—where altered diffusivity in left periventricular fibers (Dyproj/Dzassoc) correlated specifically with visuospatial/executive deficits (CDT), while the global glymphatic index (ALPS) correlated with processing speed/attention (TMT-A, SDMT)—suggests distinct neurobiological pathways (Chang Wong and Chang Chui, 2022). The former may reflect focal disconnection of white matter tracts (e.g., the superior longitudinal fasciculus) critical for the dorsal fronto-parietal network, which underpins integrative tasks like clock drawing (Shulman, 2000; Lau et al., 2024). Conversely, the association of a lower ALPS index with slower processing speed likely indicates a global physiological disruption, a phenomenon akin to the contracted hierarchical organization observed in the white matter functional connectome of patients with major depressive disorder (Yu et al., 2025). Impaired glymphatic clearance may compromise brain-wide metabolic homeostasis, thereby reducing the electrophysiological efficiency of distributed neural circuits required for rapid information processing (Iliff et al., 2012; Naganawa and Taoka, 2022). This dual-pathway hypothesis—encompassing both specific network injury and generalized functional decline—provides a framework for understanding the heterogeneity of cognitive sequelae in TBM.

DTI-ALPS as a Biomarker: Similar to its application in stroke and dementia (Liang et al., 2023), DTI-ALPS may serve as a potential biomarker for identifying TBM patients at high risk of cognitive impairment, enabling timely intervention. A reduced ALPS index may indicate an elevated risk of cognitive decline before clinical symptoms emerge, offering an opportunity for early neuroprotective strategies.

However, TBM is characterized by an acute, infection-driven inflammatory pathology, which contrasts sharply with the microglia-dominant mechanisms observed in AD (Heneka et al., 2015). This distinction highlights the need for TBM-specific interventions that combine antimicrobial treatment with glymphatic support, such as corticosteroids and TGF-β inhibitors (van Crevel et al., 2016), rather than solely mimicking Aβ-targeted approaches used in AD. Alleviating meningeal inflammation may prevent AQP4 depolarization and lymphatic fibrosis, thereby preserving glymphatic function. Although adjunctive dexamethasone has been shown to reduce TBM-related mortality (Thwaites et al., 2004), its effect on cognitive preservation remains to be fully elucidated (Prasad et al., 2016).

## 5 Limitations

First, although the DTI-ALPS technique avoids the use of conventional contrast agents (Taoka et al., 2017), its measurements may still be affected by anatomical factors such as ventricular size and white matter integrity. For instance, ventricular enlargement may lead to an underestimation of the ALPS index (Hasegawa et al., 2024), and white matter lesions may introduce potential confounding effects (Xiao et al., 2024). Future studies should incorporate multimodal imaging approaches, including structural MRI, fMRI, and dynamic contrast-enhanced MRI, to improve the accuracy and reliability of glymphatic function assessment.

Second, some confounding variables were not fully controlled in this study. These include HIV co-infection and the potential neurotoxicity of anti-tuberculosis drugs, which may influence cognitive performance (Teunissen et al., 2022). Future studies should employ stratified analyses or strict inclusion criteria to address these factors.

Third, the cross-sectional design precludes causal inferences between glymphatic dysfunction and cognitive impairment. Moreover, it should be acknowledged that the ALPS index is an indirect proxy for glymphatic function, and its validity in the TBM population lacks direct validation against established methods (e.g., intrathecal contrast-enhanced MRI). The relatively small sample size further limits the generalizability of the findings. Therefore, longitudinal studies are essential to confirm the predictive utility of the ALPS index for MCI development. Although we observed a significant association between reduced ALPS index and cognitive impairment in TBM patients, causality cannot be established. To address this, we plan to conduct longitudinal studies with larger samples, incorporating CSF biomarkers (e.g., A*β*, tau) and dynamic contrast-enhanced MRI to investigate the temporal dynamics of glymphatic dysfunction about cognitive outcomes. Additionally, we aim to explore whether elevated FA values reflect protective remodeling of specific white matter tracts or represent inflammation-related artifacts.

In conclusion, DTI-ALPS shows promise as a novel MRI-based biomarker for predicting and identifying cognitive impairment in TBM patients. It offers new insights into the underlying pathophysiological mechanisms and may provide potential therapeutic targets for interventions against secondary brain injury. However, further research is needed to establish clear causal relationships and validate its clinical utility.

## Data Availability

The raw data supporting the conclusions of this article will be made available by the authors, without undue reservation.

## Glossary

TBM: Tuberculous meningitis
MCI: Mild cognitive impairment
HCs: Healthy controls
ROC: Receiver operating characteristic
CNS: Central nervous system
CSF: Cerebrospinal fluid
AQP4: Aquaporin 4
Aβ: β-amyloid
AD: Alzheimer’s disease
PD: Parkinson’s disease
DTI-ALPS: Diffusion tensor imaging along the perivascular space
DTI: Diffusion tensor imaging
MS: Multiple sclerosis
MTB: *mycobacterium tuberculosis*
HIV: Human immunodeficiency virus
MMSE: Mini-Mental State Examination
MoCA: Montreal cognitive assessment
TMT: Trail making test
CDT: Clock drawing test
VFT: Verbal fluency test
DST: Digit Span Test
SDMT: Symbol Digit Modalities Test
MPRAGE: Magnetization prepared rapid gradient echo
FOV: Field-of-view
TR: Repetition time
FLAIR: Fluid attenuated inversion
AC-PC: Anterior commissure–posterior commissure
BET: Brain extraction tool
FA: Fractional anisotropy
ANCOVA: Analysis of covariance
CI: Confidence interval
ROC: Receiver operating characteristic
AUC: Area under the curve
LAM: Lipoarabinomannan
TLR: Toll-like receptor
BBB: Blood–brain barrier
