# Supplementary material for: Glymphatic dysfunction and cognitive impairment in tuberculous meningitis: insights from diffusion tensor imaging along the perivascular space (DTI-ALPS)
Source: Front Neurosci. 2026 Mar 6;20:1764800. doi: 10.3389/fnins.2026.1764800 (PMC13003834; doi:10.3389/fnins.2026.1764800)
Supplement: Supplementary file 1 [file Table_1.docx]

**SUPPLEMENTARY MATERIALS**

**eMethod 1**

**MRI Data Acquisition**

High-resolution three-dimensional T1-weighted magnetization-prepared rapid gradient-echo (3D-T1 MPRAGE) images were acquired at the beginning of each scanning session for anatomical registration (field of view [FOV] = 240 × 240 mm²; voxel size = 0.9 × 0.9 × 0.9 mm³; slice thickness = 0.6 mm; number of slices = 484; interslice gap = 0; repetition time [TR] = 7.9 ms; flip angle = 8°; acquisition time = 7 min 18 s).

DTI was performed using identical parameters on both scanners: FOV = 240 × 240 mm²; TR = 8000 ms; echo time = minimum; matrix = 128 × 130; diffusion encoding directions = 30; slice thickness = 5 mm; number of slices = 29; interslice gap = 0; b-values = 0 and 1000 s/mm²; acquisition time = 4 min 16 s.

T2-weighted fluid-attenuated inversion recovery (T2-FLAIR) images were obtained for all patients to exclude other brain pathologies such as stroke or tumors (TR = auto; echo time [TE] = minimum; FOV = 240 × 240 mm²; slice thickness = 5 mm; number of slices = 29; interslice gap = 0). Gadolinium-enhanced T1-weighted (Gd-T1) images were acquired for 62 patients to characterize brain lesions better, classify the type of tuberculous Meningitis, and assess ventricular enlargement.

**eMethod 2**

**Analysis of the DTI-ALPS Index**

DTI data were processed to quantify glymphatic activity using the analysis ALPS. Method, as shown in Fig. 2A. The DTI-ALPS method enables the quantification of glymphatic activity along perivascular spaces by analysing multidirectional diffusivity maps derived from DTI data. The DTI images were processed and modelled using the FMRIB Software Library (FSL, http://www.fmrib.ox.ac.uk/fsl/), following these steps:

2.1 Anterior commissure–posterior commissure (AC-PC) Alignment and Reorientation.

To correct for potential head tilt in raw images, the b0 image was rigidly aligned to a standard template using FSL’s FLIRT tool (six degrees of freedom: rotation and translation)(1), ensuring AC–PC alignment. The resulting linear deformation field was then applied to adjust the diffusion gradient directions accordingly.

2.2 Skull Stripping and Brain Extraction.

Non-brain tissues (e.g., scalp, skull) were removed using FSL’s Brain Extraction Tool (BET)(2), generating a brain mask from the b0 image for subsequent processing.

2.3 Preprocessing and Denoising.

Diffusion-weighted images (DWI) were preprocessed using MRtrix3(3) and FSL. The processing steps included: PCA-based denoising to reduce random noise, Gibbs ringing correction to suppress truncation artifacts; eddy current and motion correction using FSL’s *eddy* tool(4); and ANTs-based N4 bias field correction(5) to address B₀ inhomogeneity.

2.4 Diffusion Tensor Reconstruction.

The diffusion tensor model was fitted using FSL’s dtifit(2), producing maps of fractional anisotropy (FA) and principal directional diffusivities (Dxx, Dyy, Dzz).

2.5 ALPS Calculation.

FA maps were registered to the ICBM DTI-81 standard space atlas via T1WI using FSL, with registration accuracy verified by visual inspection. The ICBM DTI-81 atlas contains labels for projection fibers (e.g., superior and posterior corona radiata) and association fibers (e.g., superior longitudinal fasciculus) in the periventricular region. Projection and association fibers within 25–33 mm above the AC–PC line in MNI space were extracted, as this region corresponds to the x-axis direction of penetrating vessels in the deep white matter. The ALPS index and diffusivity values for projection and association fibers were computed using the method described by Taoka et al.(6).

The ALPS index is calculated based on the average values of Dxx, Dyy, and Dzz extracted within the two ROIs through the following formula:

$ALPS index=\frac{mean(D_{xxproj,}D_{xxassoc})}{mean(D_{yyproj},D_{zzassoc})}$

**eMethod 3**

**PVS Quantification**

Data analysis was performed using FreeSurfer (version 7.4.1). The software automatically delineates cortical structures, and all raw T1-weighted images underwent standard preprocessing before formal analysis. The preprocessing steps included motion correction, removal of non-brain tissue, transformation to the Talairach template for spatial normalization, intensity normalization, 3D cortical surface reconstruction, cortical inflation, and spherical mapping. These procedures were used to obtain masks of white matter, subcortical nuclei, and other relevant regions.
The Frangi vesselness filter was applied to the preprocessed T1-weighted images to estimate vessel-like structures based on the eigenvectors of the Hessian matrix for each voxel. This operation was implemented using the QIT toolkit (<https://cabeen.io/qitwiki/>), with default parameters (α = 0.5, β = 0.5), while the parameter *c* was set to half of the maximum Hessian norm. The filter was applied across multiple spatial scales (0.1–5 voxels) to identify structures with the highest likelihood of perivascular features, generating PVS probability maps.
The PVS volume within the white matter (WM), hippocampal (Hipp), and subcortical nuclear regions (basal ganglia, BG) was subsequently calculated, as shown in Fig. 2B. Total intracranial volume (TIV) was calculated from whole-brain T1-weighted image segmentation using the Computational Anatomy Toolbox (CAT12), an extension of the Statistical Parametric Mapping software package (SPM12:https://www.fil.ion.ucl.ac.uk/spm/software/spm12/). The PVS volume fraction (PVSVF; PVSVF = PVS volume/ TIV) was then calculated to eliminate interindividual variability in brain size.

**eMethod 4**

**CP Volume Measurement**

Structural T1-weighted images were processed using FreeSurfer (v7.4.1). Prior to formal brain imaging analysis, preprocessing of raw data for each subject was required, following the same steps as in the PVS section. As shown in Figure 2C, the CP within the lateral ventricles was automatically segmented from the T1-weighted images. The segmentation results underwent comprehensive visual inspection, with manual corrections applied where necessary. The final normalised CP volume was calculated by summing the bilateral CP volumes and dividing by the TIV.

**eMethod 5**

**CSF Proteomics (DIA) Analysis**

**Protein Extraction**
Frozen CSF samples were thawed on ice and suspended in protein lysis buffer (8 M urea, 1% SDS) supplemented with protease inhibitors. Samples were homogenized using a high-flux tissue grinder three times (180 s each), followed by non-contact cryogenic sonication for 30 min. After centrifugation at 16,000 g for 30 min at 8 °C, protein concentrations in the collected supernatants were determined using the bicinchoninic acid (BCA) assay (Thermo Scientific), following the manufacturer’s protocol. Protein quality was examined by SDS-PAGE.

**Protein Digestion**
A total of 100 µg of protein was resuspended in 100 mM triethylammonium bicarbonate (TEAB). Proteins were reduced with 10 mM tris(2-carboxyethyl) phosphine (TCEP) at 37 °C for 60 min and alkylated with 40 mM iodoacetamide (IAA) in the dark at room temperature for 40 min. After centrifugation at 10,000 g for 20 min at 4 °C, the pellet was resuspended in 100 µL of 100 mM TEAB. Trypsin was added at a 1:50 (enzyme:protein) ratio, and samples were incubated at 37 °C overnight.

**Peptide Desalting and Quantification**
Following digestion, peptides were dried using a vacuum concentrator, reconstituted in 0.1% trifluoroacetic acid (TFA), and desalted using HLB cartridges. Desalted peptides were dried again by vacuum concentrator and quantified using a NanoDrop One spectrophotometer (Thermo Scientific) based on UV absorbance.

**DIA Mass Spectrometry Acquisition**
Based on quantified peptide amounts, samples were analyzed using a Vanquish Neo UHPLC system coupled to an Orbitrap Astral mass spectrometer (Thermo, USA) at Majorbio Bio-Pharm Technology (Shanghai, China). Chromatographic separation was performed on a uPAC High-Throughput column (75 µm × 5.5 cm) with solvent A (water with 2% acetonitrile and 0.1% formic acid) and solvent B (80% acetonitrile with 0.1% formic acid). The LC gradient was set to 8 min. DIA data were acquired in Orbitrap DIA mode over an m/z range of 100–1700.

**Protein Identification and Quantification**
DIA raw files were processed using Spectronaut (version 19). Search parameters included a peptide length range of 7–52 amino acids, trypsin/P as the digestion enzyme, and a maximum of two missed cleavages. Carbamidomethylation of cysteine was set as a fixed modification, while methionine oxidation and N-terminal acetylation were defined as variable modifications. False discovery rate (FDR) thresholds for proteins and peptides were both set at ≤0.01, peptide confidence ≥99%, and XIC tolerance ≤75 ppm. Protein quantification was performed using the MaxLFQ algorithm.
Bioinformatic analysis was conducted on the Majorbio Cloud Platform. Differentially expressed proteins (DEPs) were identified using the R package “t-test,” with significance thresholds of fold change >1.2 or <0.83 and *p* < 0.05.

**Reference**

1. Jenkinson M, Beckmann CF, Behrens TE, Woolrich MW, Smith SM. FSL. Neuroimage 2012;62(2):782-790.

2. Smith SM. Fast robust automated brain extraction. Hum Brain Mapp 2002;17(3):143-155.

3. Tournier JD, Smith R, Raffelt D, et al. MRtrix3: A fast, flexible and open software framework for medical image processing and visualisation. Neuroimage 2019;202:116137.

4. Andersson JLR, Sotiropoulos SN. An integrated approach to correction for off-resonance effects and subject movement in diffusion MR imaging. Neuroimage 2016;125:1063-1078.

5. Tustison NJ, Avants BB, Cook PA, et al. N4ITK: improved N3 bias correction. IEEE Trans Med Imaging 2010;29(6):1310-1320.

6. Taoka T, Masutani Y, Kawai H, et al. Evaluation of glymphatic system activity with the diffusion MR technique: diffusion tensor image analysis along the perivascular space (DTI-ALPS) in Alzheimer's disease cases. Jpn J Radiol 2017;35(4):172-178.
